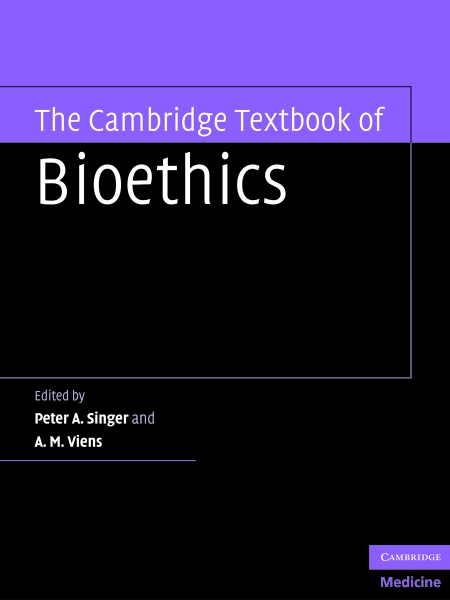# Bioethics Grows Up

**DOI:** 10.1371/journal.pbio.0060095

**Published:** 2008-04-08

**Authors:** Arthur Caplan

## Abstract

Arthur Caplan reviews*The Cambridge Textbook of Bioethics*.

When I taught my first course in bioethics to first-year students at Columbia University's College of Physicians and Surgeons in the spring semester of 1981, bioethics was still in its formative years. There were scant few textbooks around and even fewer anthologies, and I could not assume that any of my students had ever read anything by a bioethicist or about bioethics. The key institutions in the field at that time, the Hastings Center, then in Hastings-on-Hudson, New York, and the Kennedy Institute at Georgetown University in Washington, DC, were barely over a decade old, and only one journal devoted to bioethics had been publishing for a significant period of time. Most major medical and biomedical journals were wary of publishing pieces on ethics, because the editors did not think that articles on a soft and mushy subject such as ethics were appropriate for journals of medicine and science. An instructor in those days really had to scramble to find and assemble the best writings and be ready to incur a hefty Xeroxing bill.

These days when I organize a class, I can expect that nearly every student at the University of Pennsylvania School of Medicine will have taken at least one course in bioethics as an undergraduate. Some will even have had a course or some exposure to the field in high school. Today, the challenge isn't finding material to teach but choosing from among the ever-growing abundance of readers, textbooks, articles, journals, and guides on bioethics. At least a dozen impressively hefty anthologies have appeared in the last three years from highly regarded publishing houses, edited by readily recognizable names in the field. They are fighting for sales in what has become a lucrative bioethics market. In fact, there are so many books out there now that new publications have begun to specialize, in the hope of minimizing competition by carving out a sub-niche. Some readers and texts target clinicians, researchers, undergraduates, or high school students while others take explicitly religious approaches, or explore bioethics from feminist, international, or legal perspectives.

Singer and Viens' *The Cambridge Textbook of Bioethics* does not take the specialty turn. It is big—554 pages. It is broad, with sections on end-of-life care, genetics, research ethics, the ethics of health systems, clinical ethics, and religious perspectives on bioethics. It is brassy, with articles on neuroethics, alternative medicine and ethics, anesthesiology ethics, and aboriginal bioethics. And it is… Canadian. A huge number of the contributors, more than have ever been assembled inside one cover, come from Canada, no doubt reflecting Singer and Viens' ties to the Joint Center for Bioethics at the University of Toronto.

Some of the articles that appear in *The Cambridge Textbook of Bioethics* are new or at least have been updated from earlier published versions. All of them are concise, easy to read, and well-referenced. But these virtues are also part of the problem with this collection.

The book is not suitable for undergraduates. They will not have the background to engage most of the subject matter and would need fewer topics and more in-depth articles. Similarly, the book lacks enough specialized content to engage biomedical researchers. Although there is a section on research ethics, it is a disjointed overview of the topic. There is not enough substance in any particular article to help a researcher work through specific issues that are especially timely these days, such as subject recruitment, payments to researchers and subjects, emergency research, or research involving vulnerable populations such as children, elderly, or the mentally ill. Researchers would also benefit from more information on how to terminate a clinical trial that is not going well and the duties of institutional review boards (IRBs) and data safety and monitoring committees (DSMs) in overseeing clinical trials being conducted in many locations in many nations.

The editors say that the book is aimed at practicing clinicians and health care managers, which I agree would be the best audience, because the large content area does overlap the interests of these groups. However, in my experience, the only way to really engage practitioners and managers with bioethics is through case studies. Practitioners live in a sea of cases—that is how they are taught and that is the medium in which they operate. The same is true for those managing in health care settings and systems.

Yet, *The Cambridge Textbook of Bioethics* is not a casebook. As such, clinicians are not likely to crack its covers unless they are motivated to do so by a particular case they are trying to solve. But they will find relatively few cases. Even if the book is used in conjunction with case material produced from other sources, the curious, puzzled, or panicking clinician or manager may find that the kind of appetizer-sized portions available in this book are not going to sate an appetite for in-depth discussion.

Despite these problems, *The Cambridge Textbook of Bioethics* is a very strong collection. It has skilled and knowledgeable editors, high-caliber contributors, and contributions that are uniformly of good quality.

For example, the article on emergency and trauma medicine ethics by Arthur B. Sanders does a very nice job addressing the problem of how to manage patients who are often unconscious, who do not choose their doctors, for whom time is of the essence, and who sometimes arrive without family or friends. Sanders notes that the presumption for diagnosis and treatment dominates emergency care and that when in doubt, doctors must err on the side of treatment including resuscitation. He also notes that ER doctors have the right to stop resuscitation efforts when they judge the patient to be unresponsive regardless of protests by family or friends. Autonomy can be trumped by a clinical determination of futility—a principle that has obvious and important implications well beyond emergency and trauma care.

Similarly, Sidney Bloch and Stephen A. Green take the reader through the challenging case of a young woman who, after the birth of her first baby, begins exhibiting odd behavior and mildly paranoid thinking. The woman denies any thoughts of suicide, and her husband doesn't want her put into a psychiatric facility where, in his view, only severely mentally ill patients reside. While respecting the need to respect her autonomy, Bloch and Green make the useful suggestion that short of committing the new mother to involuntary treatment against her and her husband's wishes, it may be possible to arrange for her rigorous and constant supervision by family and friends, so that any further slippage in her behavior or improvement can be detected and verified while keeping her and her baby safe. Managing patient care by building trust alongside respect for autonomy is highly useful advice in managing difficult cases such as this.

So, as these examples reveal, there is much of value in this book. Which raises the question: if overly broad scope and lack of depth are the book's major failings, has bioethics now outgrown the ability of any single reader or text to adequately capture the entire field? I think it has.

When I began teaching, there was no agreed-upon set of topics or issues—no canon—that defined the field. An instructor simply hunted around for articles on topics of interest, and that was the syllabus. Today, bioethics has exploded in parallel with the explosion in clinical medicine, the health professions, and the biomedical sciences. The literature in the field is enormous. There are certainly core topics that now constitute the canon of the field, and Singer and Viens have nailed most of them. But there is too much writing in too many places by too many authors from too many fields to try and capture the whole field in a single anthology or text. Add to this explosion in the literature the fact that most of what is written is available free on the Web or at least is accessible in a PDF file within a year of its publication, and you start to see that the writing is on the wall for the general anthology in bioethics.

The future of the field is specialization—in the way research is done, teaching is offered, and books and articles are written. Future undergraduates, health care students, and budding researchers are not going to gain access to the field by sampling a smorgasbord of articles. The future of bioethics depends upon its ability to engage students and their teachers who have a focused range of concerns, such as research involving human subjects, research using animals, synthetic biology, critical care, organ and tissue transplantation, conflict of interest, vaccine ethics, or neuroethics.

With bioethics entering a new phase, the large, general anthology has become a lumbering creature that is not nimble enough to keep up with a rapidly evolving environment. The future, despite the high-quality work insightfully collected in *The Cambridge Textbook of Bioethics*, belongs to those who can meet what will be an increasing and unremitting demand for specialized, focused work in bioethics.

**Figure:**